# Impact of COVID-19 Lockdown on the Nasopharyngeal Microbiota of Children and Adults Self-Confined at Home

**DOI:** 10.3390/v14071521

**Published:** 2022-07-12

**Authors:** Muntsa Rocafort, Desiree Henares, Pedro Brotons, Cristian Launes, Mariona Fernandez de Sevilla, Victoria Fumado, Irene Barrabeig, Sara Arias, Alba Redin, Julia Ponomarenko, Maria Mele, Pere Millat-Martinez, Joana Claverol, Nuria Balanza, Alex Mira, Juan J. Garcia-Garcia, Quique Bassat, Iolanda Jordan, Carmen Muñoz-Almagro

**Affiliations:** 1 Institut de Recerca Sant Joan de Déu (IRSJD), Hospital Sant Joan de Deu, Esplugues de Llobregat, 08950 Barcelona, Spain; muntsa.rocafort@sjd.es (M.R.); desiree.henares@sjd.es (D.H.); pedro.brotons@sjd.es (P.B.); cristian.launes@sjd.es (C.L.); mariona.fernandez@sjd.es (M.F.d.S.); victoria.fumado@sjd.es (V.F.); alba.redin@sjd.es (A.R.); maria.mele@sjd.es (M.M.); joana.claverol@sjd.es (J.C.); juanjose.garciag@sjd.es (J.J.G.-G.); yolanda.jordan@sjd.es (I.J.); 2 CIBER of Epidemiology and Public Health (CIBERESP), Instituto de Salud Carlos III, 28029 Madrid, Spain; ibarrabeig@gencat.cat (I.B.); mira_ale@gva.es (A.M.); quique.bassat@isglobal.org (Q.B.); 3 Medicine Department, Universitat Internacional de Catalunya, Sant Cugat, 08195 Barcelona, Spain; 4 Pediatrics Department, Hospital Sant Joan de Déu, Universitat de Barcelona, Esplugues de Llobregat, 08950 Barcelona, Spain; 5 Epidemiological Surveillance Unit, Department of Health, Generalitat de Catalunya, 08907 Barcelona, Spain; 6 ISGlobal, Hospital Clínic-Universitat de Barcelona, 08036 Barcelona, Spain; sara.arias@isglobal.org (S.A.); pere.millat@isglobal.org (P.M.-M.); nuria.balanza@isglobal.org (N.B.); 7 Centre for Genomic Regulation (CRG), The Barcelona Institute of Science and Technology, 08003 Barcelona, Spain; julia.ponomarenko@crg.eu; 8 Department of Experimental and Health Sciences, Universitat Pompeu Fabra (UPF), 08002 Barcelona, Spain; 9 Department of Health and Genomics, Center for Advanced Research in Public Health, Fundacion para el Fomento de la Investigacion Sanitaria y Biomedica de la Comunitat Valenciana (FISABIO), 46020 Valencia, Spain; 10 Institució Catalana de Recerca i Estudis Avançats (ICREA), 08010 Barcelona, Spain; 11 Centro de Investigação em Saúde de Manhiça (CISM), Manhiça Maputo 1929, Mozambique

**Keywords:** nasopharyngeal microbiota, children, adults, COVID-19, SARS-CoV-2

## Abstract

The increased incidence of COVID-19 cases and deaths in Spain in March 2020 led to the declaration by the Spanish government of a state of emergency imposing strict confinement measures on the population. The objective of this study was to characterize the nasopharyngeal microbiota of children and adults and its relation to SARS-CoV-2 infection and COVID-19 severity during the pandemic lockdown in Spain. This cross-sectional study included family households located in metropolitan Barcelona, Spain, with one adult with a previous confirmed COVID-19 episode and one or more exposed co-habiting child contacts. Nasopharyngeal swabs were used to determine SARS-CoV-2 infection status, characterize the nasopharyngeal microbiota and determine common respiratory DNA/RNA viral co-infections. A total of 173 adult cases and 470 exposed children were included. Overall, a predominance of *Corynebacterium* and *Dolosigranulum* and a limited abundance of common pathobionts including *Haemophilus* and *Streptococcus* were found both among adults and children. Children with current SARS-CoV-2 infection presented higher bacterial richness and increased *Fusobacterium*, *Streptococcus* and *Prevotella* abundance than non-infected children. Among adults, persistent SARS-CoV-2 RNA was associated with an increased abundance of an unclassified member of the Actinomycetales order. COVID-19 severity was associated with increased *Staphylococcus* and reduced *Dolosigranulum* abundance. The stringent COVID-19 lockdown in Spain had a significant impact on the nasopharyngeal microbiota of children, reflected in the limited abundance of common respiratory pathobionts and the predominance of *Corynebacterium*, regardless of SARS-CoV-2 detection. COVID-19 severity in adults was associated with decreased nasopharynx levels of healthy commensal bacteria.

## 1. Introduction

Coronavirus Disease 2019 (COVID-19), caused by the Severe Acute Respiratory Syndrome Coronavirus 2 (SARS-CoV-2), first emerged in Wuhan, China, in December 2019 [1] and subsequently spread globally as a pandemic. Since then, new cases of SARS-CoV-2 have rapidly increased worldwide leaving COVID-19 as one of the most devastating pandemics in human history [2].

As a result of the unprecedented increase in cases and deaths and to prevent the collapse of the health system, a state of emergency was declared by the Spanish government from March to June 2020, enforcing strict confinement for most of the population and the closure of most business and all leisure, cultural and educational activities [3]. Measures implemented to mitigate SARS-CoV-2 transmission also included social distancing, frequent hand washing and individual protection equipment, which have been proven useful to prevent infectious diseases spread by human contact [4,5]. 

These containment measures aimed to mitigate SARS-CoV-2 transmission but appeared to have a concomitant effect on a decline in the incidence of other respiratory infectious diseases. In particular, sustained decreases in invasive diseases due to common respiratory pathobionts such as *Streptococcus pneumoniae*, *Haemophilus influenzae* and *Neisseria meningitidis* were reported across all ages in Catalonia (Northern Spain) and other regions across Europe [6]. 

The nasopharyngeal cavity, a primary gateway for SARS-CoV-2 entry, harbors a microbial ecosystem in constant interplay with the host immune system and the environment, ultimately influencing health and disease [7,8,9]. In recent years, many respiratory diseases caused by pathobionts have been related with an imbalance of the microbiota of the upper respiratory tract [10]. Hence, not only bacterial pathogens, but also bacterial commensals and viral agents, are being recognized as key players in infection and disease pathogenesis. Moreover, children’s nasopharyngeal microbiota is highly dynamic during the first years of life, and it is known to be heavily affected by environmental factors such as kindergarten attendance as well as antibiotic usage, among others [11,12].

To date, little is known about the role of the nasopharyngeal microbiota in the global decrease in bacterial invasive diseases concomitant to the adoption of social distancing measures and use of facial masks to combat the COVID-19 pandemic, as well as to its relation to SARS-CoV-2 infection in children and adults. The first objective of this study was to characterize the nasopharyngeal microbiota of adults either convalescent or recovered from a previous RT-PCR-confirmed COVID-19 episode and of all contact children in an unprecedented scenario of strict home quarantine during the 2020 pandemic lockdown. The second objective was to assess the relationship between the nasopharyngeal microbiota composition and current SARS-CoV-2 RNA detection in exposed children as well as SARS-CoV-2 RNA’s persistent detection and COVID-19 disease severity in primary adult cases.

## 2. Materials and Methods 

### 2.1. Design and Study Population

The study was conducted by researchers of the University Hospital Sant Joan de Déu (HSJD) in Barcelona, Spain. Details of participants and data and sample collection procedures have been described in previous publications [13]. In brief, we prospectively enrolled volunteer families that included: (i) one adult parent who was the first family member reported as positive by SARS-CoV-2 RT-PCR at least 15 days before sample collection, and (ii) at least one child aged less than 15 years co-habiting in the same household. Enrollment targeted family households located in the health region of the Metropolitan Area of Barcelona and spanned from 28 April to 3 June 2020. 

### 2.2. Inclusion and Exclusion Criteria

A total of 410 families that participated in the household prevalence study [13] were initially considered for the study. Only individuals with a valid SARS-CoV-2 RT-PCR result and enough volume of nasopharyngeal swab sample for microbiota characterization were included. Additionally, samples with less than 10,000 reads/sample after sequencing data quality filtering and contamination removal, were excluded from the study. 

### 2.3. Microbiological Methods

Nasopharyngeal swabs were collected from all eligible participants. Each sample was aliquoted into 2 subsamples: one was processed for multiple respiratory virus detection at HSJD, and another was transferred to the laboratory of Centro de Regulación Genómica (Barcelona, Spain) for SARS-CoV-2 RT-PCR testing as well as for 16S rRNA gene amplification and sequencing. Samples were introduced into storage tubes (Micronics) with transport medium for pathogen inactivation (Zymo DNA/RNA Shield Lysis Buffer™; Zymo Research, Freiburg, Germany). 

SARS-CoV-2 RT-PCR was performed according to the CDC-006-00019 protocol released on 30 March 2020 that includes detection of N1 or N2 SARS-CoV-2 genes and RNase P human gene as internal control. Presence of other viral respiratory infections in nasopharyngeal samples was tested by Allplex™ Respiratory Panels Assays 1, 2 and 3 (Seegene Inc., Seoul, Korea) targeting 16 viruses (rhinovirus, enterovirus, adenovirus, bocavirus, coronavirus, metapneumovirus, respiratory syncytial virus types A and B, influenza virus types A and B, and parainfluenza virus types 1, 2, 3 and 4). Detailed protocols for SARS-CoV-2 and other respiratory viruses’ detection have been previously published. 

### 2.4. DNA Extraction and 16S rRNA Sequencing 

DNA was extracted from nasopharyngeal swabs using the ZymoBIOMICS 96 MagBead NA Kit (Zymo Research, Freiburg, Germany) and following manufacturer’s instructions. The extraction tubes were agitated using Tissue lyser II (Qiagen, Hilden, Germany) at 30 Hz/s for 10 min. 

The 16S rRNA gene amplicons were produced using the V3-V4 region specific degenerate primers: Forward 5′-TCGTCGGCAGCGTCAGATGTGTATAAGAGACAGNCCTACGGGNGGCWGCAG-3′ and Reverse 5′-GTCTCGTGGGCTCGGAGATGTGTATAAGAGACAGNGACTACHVGGGTATCTAATCC-3′. PCR was performed in a 25 μL final volume with a 0.2-μM primer concentration under the following cycling conditions: 3 min at 95 °C, 35 cycles of 30 s at 95 °C, 30 s at 55 °C, and 30 s at 72 °C, and a final elongation step of 5 min at 72 °C. The PCR products were purified using AMPure XP beads (Beckman Coulter, Nyon, Switzerland), with a 0.9× ratio according to manufacturer’s instructions. Nextera XT v2 barcoded adapters were used as part of the library preparation protocol resulting in final ~460 bp amplicons. Twenty-five μL of all barcoded PCR products were purified with SequalPrep normalization kit (Invitrogen, ThermoFisher Scientific, Waltham, MA, USA), according to the manufacturer’s protocol. 

The final library pool was quantified by qPCR using Kapa library quantification kit for Illumina Platforms (Kapa Biosystems, Sigma Aldrich, Saint Louis, MO, USA) on an ABI 7900HT real-time cycler (Applied Biosystems, ThermoFisher Scientific, Waltham, MA, USA) and sequenced using Illumina MiSeq (2 × 300 bp) on v3 chemistry with a loading concentration of 15 pM. In total, 10% of PhIX control libraries were used to increase the diversity of the sequenced samples and negative (extraction and PCR) controls were also included in the final library pool.

### 2.5. Bioinformatics Analysis 

Quality of sequencing data was assessed with FASTQC toolkit. All further bioinformatic data analyses were conducted using R programming language. Amplicon sequence variants (ASVs) were binned and quantified using the DADA2 package [14] in R using the RDP set 16 taxonomic reference database [15]. As part of the DADA2 pipeline, prior to paired end read merging, forward and reserve reads were trimmed at positions 10/10 and 280/230 starting and ending positions, respectively, to improve read quality.

Alpha diversity metrics (Observed and Chao1 for richness, and Shannon and Inverse Simpson for diversity) were calculated at the ASV level after rarefying samples at 10,000 reads using Phyloseq package [16]. Non-rarefied samples were utilized for the rest of analyses either at the ASV level or at the genus level by collapsing reads assigned to the same bacterial genera using Phyloseq package [16]. Beta diversity analyses included ordination analyses using PCoA and Bray–Curtis ecological distance over non-filtered bacterial ASV’s relative abundance matrix. Differences in overall composition were tested with PERMANOVA using Adonis2 function from Vegan package [17] in R. Differential abundance testing of bacterial genera was conducted using the ANCOM-BC [18] test for raw reads. All comparisons between groups were internally validated using randomly selected subgroups of same number of individuals to overcome bias in group sample size.

Decontam package [19] was used to identify potential kit reagent and environmental contaminants in the 25 sequenced negative controls (13 extraction and 12 PCR controls) so they could be further removed from nasopharyngeal participant samples. Additionally, reads that were not assigned to kingdom bacteria or were classified as such but no further taxonomic resolution was reached were excluded. Reads mapping against the human reference genome (hg38) were also removed from the sequencing dataset. Lastly, only nasopharyngeal samples with a minimum sequencing coverage of 10,000 reads and a valid SARS-CoV-2 RT-PCR result were considered.

### 2.6. Statistical Analysis

Categorical variables were assessed with the Chi-square or Fisher exact test (if <25% of cells with <5 values) for description of the study population. Continuous variables were described as means and standard deviations (SD) or as median and interquartile range (IQR) values and were further analyzed using ANOVA (for normally distributed samples) or Kruskal–Wallis test (for non-normally distributed samples). 

## 3. Results 

### 3.1. Epidemiological and Clinical Characteristics of Participants

Six hundred and forty-three individuals, 173 adult cases and 470 exposed children, were included in the study. The median age of the children was 4.4 years [IQR: 2.6–7.5] and 48.1% of them were female (Table 1). Among the pediatric group, 45 (9.6%) children tested positive for SARS-CoV-2 RNA. All positive children were asymptomatic (*n* = 447, 95.5%) or had mild respiratory symptoms (*n* = 21, 4.5%) Both SARS-CoV-2 positive and negative groups of children were similar in age, gender, time elapsed since primary adult case diagnosis by SARS-CoV-2-RT-PCR, body temperature, presence of respiratory symptoms, as well as antibiotic and probiotic use within the previous 3 months before sample collection. SARS-CoV-2 positive children showed a higher prevalence of respiratory virus co-infection (44.4 vs. 22.4%, *p* = 0.002), including higher rates of rhinovirus/enterovirus infection (40.0 vs. 16.7%, *p* < 0.001) and parainfluenza virus type 1 (6.7 vs. 0.7%, *p* = 0.013) (Table 1). 

A higher incidence of rhinovirus/enterovirus among SARS-CoV-2 positive children was observed irrespective of age range, although this was more evident among those aged younger than 5 years (Supplementary Tables S1 and S2).

The median age of adult participants was 39.9 years [IQR: 35.9–44.4] and 36.4% were female (Table 2). All adults had already been diagnosed with SARS-CoV-2 infection before study enrollment with a mean time lag between diagnosis of infection and new RT-PCR test of 53 days [IQR: 44–61]. At the time of study enrollment, twenty adults (11.8%) reported active respiratory symptoms, which included: dyspnea (*n* = 10); dyspnea and anosmia (*n* = 1); dyspnea and asthenia (*n* = 1); dyspnea, anosmia and dysgeusia (*n* = 1); dyspnea and cough (*n* = 1) and cough (*n* = 4). Forty-seven (27.2%) adults showed a persistent positive result for SARS-CoV-2 RNA detection. Thirty-six adults reported past hospitalization because of COVID-19 (20.8%) with a median length of stay of 7 days [IQR: 5.0–10.3].

Both SARS-CoV-2 RNA persistent and non-persistent detection groups were similar in age, gender, time elapsed since primary diagnosis by SARS-CoV-2-RT-PCR, body temperature, as well as antibiotic and probiotic use within the 3 months before sample collection. Adults with persistent positive SARS-CoV-2-RT-PCR (*n* = 47) showed a higher prevalence of respiratory symptoms (22.2 vs. 8.0%, *p* = 0.023) as well as a higher rate for viral co-infection (14.9 vs. 2.4%, *p* = 0.006), specifically for rhinovirus/enterovirus (12.8 vs. 2.4%, *p* = 0.019) (Table 2). Using past COVID-19-related hospitalization as a surrogate for disease severity, adults that required hospital admission showed a higher male proportion (69.4 vs. 27.7%, *p* < 0.001), older median age (44.2 [IQR: 36.9–48.4] vs. 39 years [IQR: 35.9–42.9], *p* < 0.001) and higher use of antibiotics within the previous 3 months before sample collection (85.3 vs. 24.0%, *p* < 0.001) (Table 2). No differences were found for viral co-infection between those adults who required hospitalization and those who did not.

### 3.2. Nasopharyngeal Microbiota of Children and Adults Dominated by Corynebacterium with Limited Relative Abundance of Common Pathobionts

**Figure 1:**
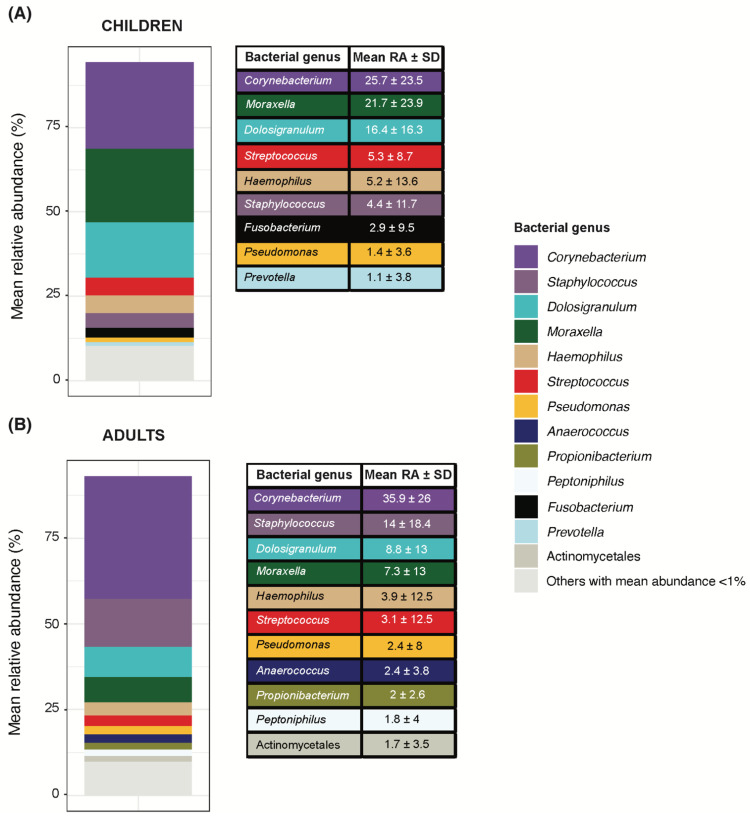
Bacterial genera composition in the nasopharynx of children and adults. Bacterial genera were filtered by a minimum of 0.01% relative abundance in at least 10% of samples within each study group (children in (**A**) and adults in (**B**)). Only bacterial genera with a mean abundance > 1% are shown in the table ranked from most to least abundant in each group. Same genera are properly identified by color coding as shown in the legend and kept consistent in the two groups. Bacterial genera whose mean relative abundance was <1% are grouped into “others”.

Despite overall significant differences in nasopharyngeal microbiota composition between adults and children at the Amplicon Sequence Variant (ASV) level (PERMANOVA R^2^ = 2.5%/*p* < 0.01), the mean relative abundance ranking of bacterial genera per group shared the top taxa between them (Figure 1). *Corynebacterium* was the topmost abundant bacterial genus with a mean relative abundance above 25% in both adults and children (25.7% and 35.9%, respectively). 

Beyond *Corynebacterium*, children’s nasopharyngeal microbiota was dominated by *Moraxella* (21.7%) and *Dolosigranulum* (16.4%), altogether accounting for an overall mean relative abundance of 63.8% (Figure 1A). In contrast, bacterial genera including common respiratory pathobionts in children such as *Streptococcus* (5.3%), *Haemophilus* (5.2%) and *Staphylococcus* (4.4%), among others, showed comparatively lower relative abundance, with mean values around 5% (Figure 1A). This trend of the high mean abundance of *Corynebacterium*, *Moraxella* and *Dolosigranulum,* accompanied by low abundance of common pediatric pathobionts was consistently observed for both pediatric age ranges (Supplementary Figure S2). 

*Corynebacterium’s* mean relative abundance in adults was 35.9%, with *Staphylococcus* (14%)*, Dolosigranulum* (8.8%) and *Moraxella* (8.8%), among others, following far behind (Figure 1B). 

### 3.3. SARS-CoV-2 RNA Detection in Children Associated with Higher Bacterial Richness and Higher Fusobacterium, Streptococcus and Prevotella Abundance

**Figure 2:**
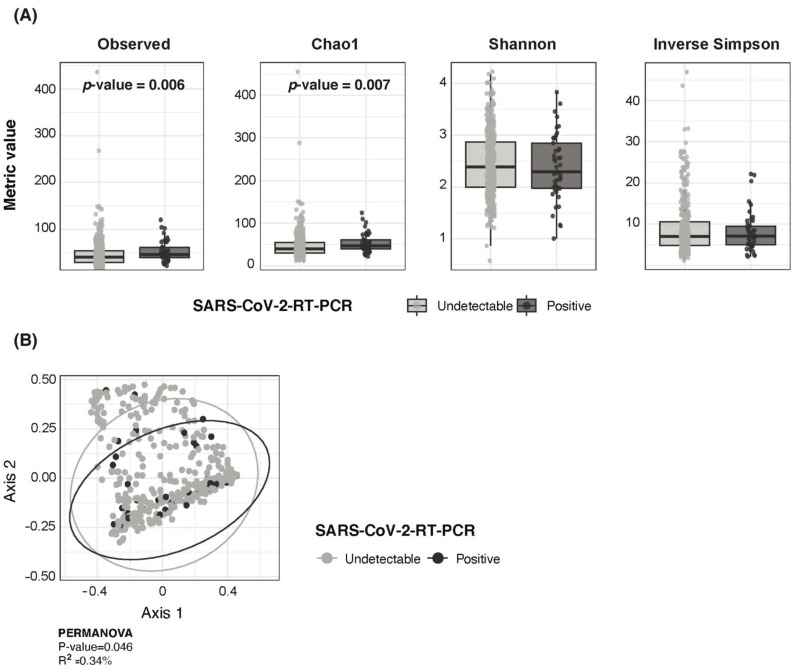
Pediatric SARS-CoV-2 infection associated with higher bacterial richness but similar diversity and overall microbiota composition. (**A**) Boxplots showing richness (Observed and Chao 1) diversity (Shannon and Inverse Simpson) metrics between SARS-CoV-2 RNA detection groups in children. (**B**) PCoA ordination analysis on Bray–Curtis ecological distance matrix showing distribution of SARS-CoV-2 positive and negative pediatric samples.

Children with a positive detection of SARS-CoV-2 RNA showed higher bacterial richness but similar bacterial diversity than children with no RNA detection (Figure 2A). At the ASV level, there were no major differences in overall nasopharyngeal bacterial composition by SARS-CoV-2 RNA detection status (PERMANOVA R^2^ = 0.34%/*p* = 0.046) (Figure 2B). 

**Figure 3:**
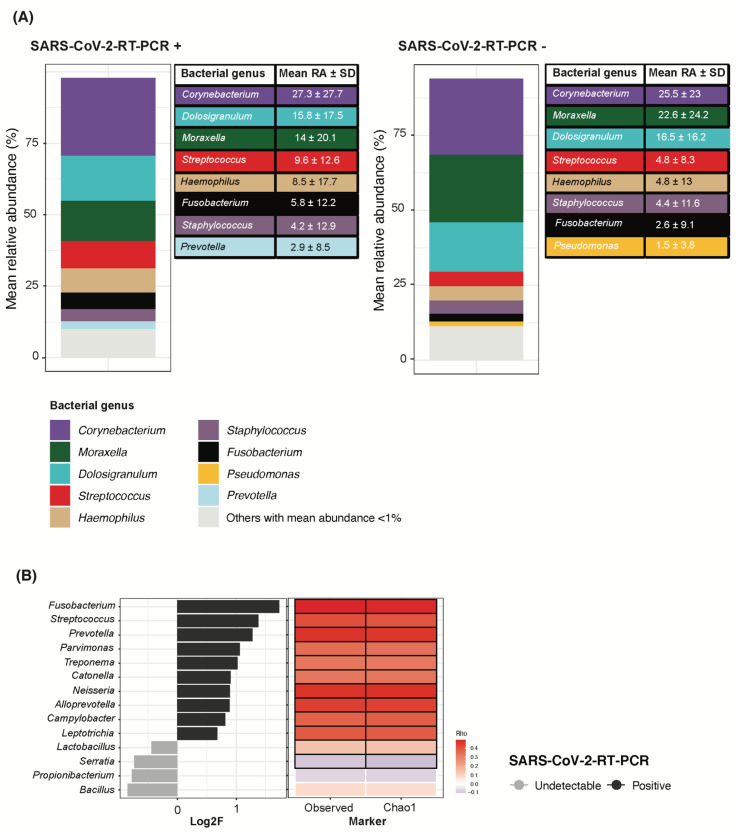
SARS-CoV-2 RNA detection in children associated to increased *Fusobacterium*, *Streptococcus* and *Prevotella* abundance, among others. (**A**) Bacterial genera were filtered by a minimum of 0.01% relative abundance in at least 10% of samples within each study group based on the SARS-CoV-2 RNA detection result. Only bacterial genera with a mean abundance > 1% are shown in the table ranked from most to least abundant in each group. Same genera are properly identified by color coding as shown in the legend and kept consistent in the two groups. Bacterial genera whose mean relative abundance was <1% are grouped into “others”. (**B**) Differential abundance analysis on bacterial genera. Log2F is shown along the *X*-axis and differential genera are colored based on the SARS-CoV-2 RNA detection group they relate to. On the right, Spearman correlations are shown between each differential bacterial genera and markers for bacterial richness. Red stands for positive correlation and blue for negative correlation. Significant Rho values are marked with a black square.

Taxonomic composition at the genus level showed that *Corynebacterium* remained the topmost abundant bacterial genus in both SARS-CoV-2 RNA detection groups with mean abundances of 27.3% and 25.5% for RNA positive and negative children, respectively. *Moraxella* and *Dolosigranulum* completed the three topmost abundant taxa (Figure 3A). A limited abundance of common pathobionts including *Streptococcus*, *Haemophilus* and *Staphylococcus* was found irrespective of SARS-CoV-2 RNA detection (Figure 3A). However, a differential abundance analysis between SARS-CoV-2 RNA detection groups showed that higher *Fusobacterium*, *Streptococcus* and *Prevotella* abundance, among others, was associated with a positive detection of SARS-CoV-2 RNA (Figure 3B). In line with higher bacterial richness, all bacterial genera found to have a higher abundance with SARS-CoV-2 RNA detection were positively correlated to both the observed richness and Chao1 index (Figure 3B). 

### 3.4. Adult COVID-19 Severity Associated to Higher Staphylococcus and Lower Dolosigranulum Abundance

**Figure 4:**
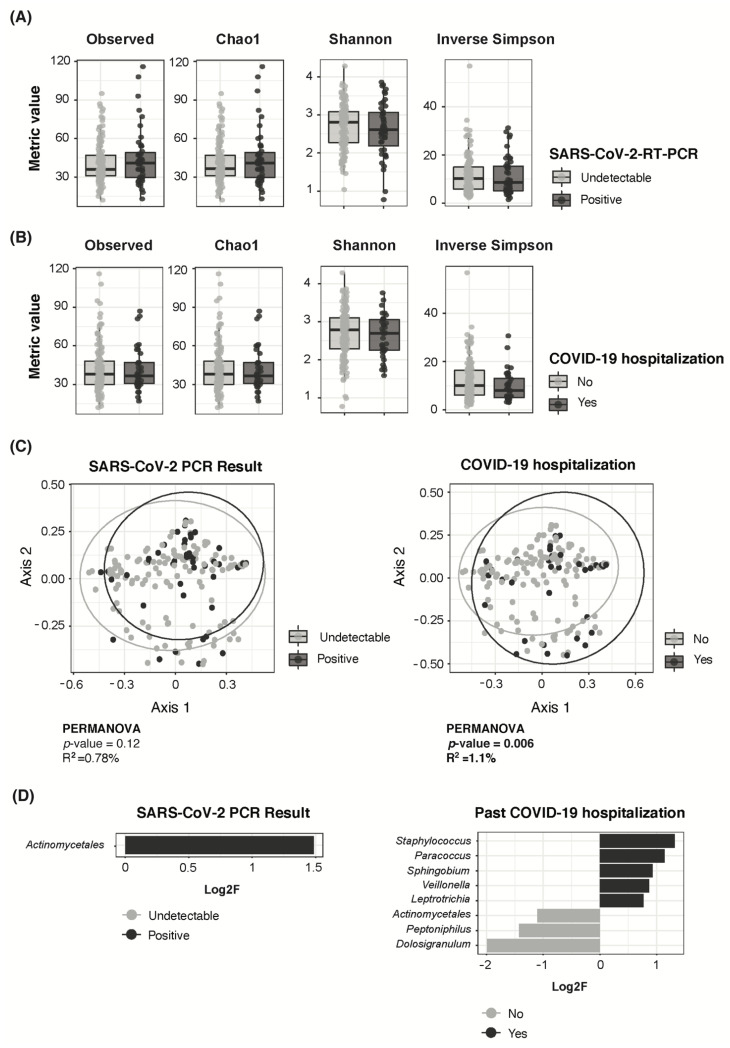
Nasopharyngeal microbiota composition in adults according to SARS-CoV-2 RNA persistence and COVID-19 severity. (**A**) Boxplots showing richness (Observed and Chao 1) and diversity (Shannon and Inverse Simpson) metrics between SARS-CoV-2 RNA persistence groups in adults. (**B**) Boxplots showing richness (Observed and Chao 1) and diversity (Shannon and Inverse Simpson) metrics by history of COVID-19-related hospitalization in adults. (**C**) PCoA ordination analysis on Bray–Curtis ecological distance matrix showing distribution of SARS-CoV-2 positive and negative adult samples on the left, and by history of COVID-19 hospitalization on the right. (**D**) Differential abundance analysis on bacterial genera by SARS-CoV-2 RNA persistence (left) or by history of COVID-19-related hospitalization (right). Log2F is shown along the *X*-axis and differential genera are colored based on the group they relate to.

The adult population did not show significant differences in bacterial richness or diversity, either by SARS-CoV-2 RNA persistence (Figure 4A) or past hospitalization due to COVID-19 (Figure 4B). SARS-CoV-2 RNA persistence had no significant effect on overall nasopharyngeal bacterial composition at the ASV level (PERMANOVA R^2^ = 0.78%/*p* = 0.12) (Figure 4C). Past COVID-19-related hospitalization had a significant yet limited effect (PERMANOVA R^2^ = 1.1%/*p* = 0.006) (Figure 4C).

Taxonomic composition analyses showed that the top three most abundant bacterial genera consistently included *Corynebacterium*, *Staphylococcus* and *Dolosigranulum*, irrespective of SARS-CoV-2 RNA persistence or past COVID-19-related hospitalization (Supplementary Figure S2). A differential abundance analysis showed that SARS-CoV-2 RNA persistent detection in adults was associated with increased abundance of an unclassified member of the *Actinomycetales* order compared with previously infected adults with current undetectable SARS-CoV-2 RNA levels (Figure 4D). Past COVID-19-related hospitalization was associated to a higher abundance of *Staphylococcus* as well as to a lower abundance of *Dolosigranulum*, among others, compared to non-hospitalized adults (Figure 4D). 

## 4. Discussion 

This study evidenced the low abundance of common pathobionts and high *Corynebacterium* abundance in the nasopharynx of children and adults quarantined in their family households during the COVID-19 lockdown in Spain.

Children showed a remarkably high abundance of *Corynebacterium,* closely followed by *Moraxella* and *Dolosigranulum*, with a significant minor contribution of common pathobionts including genera *Streptococcus* and *Haemophilus*. These findings contrast with pre-pandemic studies from our group, among others, in which *Haemophilus*, *Moraxella* and *Streptococcus* were the topmost abundant genera in the nasopharynx of children from the same geographical area, irrespective of their respiratory health status, and were followed far behind by *Dolosigranulum* and *Corynebacterium* [10,20]. For example, Henares et al. reported considerably higher mean abundances of *Streptococcus* (33.1%, 12.1% and 15.99%) *Haemophilus* (24.8%, 37.9% and 28.8%) and *Moraxella* (32.0%, 37.9% and 35.7%) in children with Invasive Pneumococcal Disease (IPD), upper respiratory tract infection or healthy controls, respectively, while a minor presence of *Corynebacterium* (0.53%, 1.26% and 0.97%) and *Dolosigranulum* (1.3%, 2.1% and 9.3%) was observed across the three groups [10].

Children’s nasopharyngeal microbiota is highly unstable, especially during the first year of life, and initial colonizers such as *Staphylococcus*, *Dolosigranulum* and *Corynebacterium* are gradually replaced by common pathobionts including *Moraxella*, *Streptococcus* and *Haemophilus* [21,22]. The mode of delivery, short duration of breastfeeding, kindergarten attendance, concomitant viral infections and antibiotic use have been associated with the loss of protective bacteria, such as *Corynebacterium* and *Dolosigranulum,* and enrichment of pathobionts [11,23,24,25,26,27]. Remarkably, during the COVID-19 pandemic, alongside the limited social interactions and the personal protective measures, many of these factors were reported to be altered, including the reduced outpatient prescription of common antibiotics [28,29].

Additionally, the topmost abundant bacterial genera in our dataset, *Dolosigranulum* and *Corynebacterium*, have been identified as protective bacteria negatively correlated with nasal/nasopharyngeal colonization by *Staphylococcus aureus* or *Streptococcus pneumoniae* [30,31]. Specifically, *Dolosigranulum pigrum* was reported to be underrepresented in the nasopharynx of children with IPD compared to healthy children [10,20]. Similarly, different studies with adult populations reported an increased abundance of *Corynebacterium* and *Dolosigranulum* in healthy adults compared with adults with community-acquired pneumonia [7,32,33]. 

Altogether, our results suggest that the dynamics of transmission and nasopharyngeal colonization of common pathobionts and commensal bacteria changed substantially among children during the COVID-19 pandemic lockdown. A distinct nasopharyngeal microbiota profile has been identified in quarantined children that resembles that of the adult’s microbiota in the pre-COVID-19 era [32]. Reduced outdoor interaction between children along with intensive and close contact between household members during home quarantine could have contributed to explain this shift in children’s nasopharyngeal microbiota composition. The significant decline in infectious diseases caused by common respiratory pathobionts such as *Streptococcus pneumoniae* and *Haemophilus influenzae*, among others, reported in our geographical area [6], may also be associated to the impact of the lockdown on the nasopharyngeal microbiota. Nonetheless, given the complex interaction network between the microbiota and the host immune system, and the uncertainty of the long-term effects of COVID-19 on the human microbiota [34], we cannot be sure if these observed short-term alterations on the children’s nasopharyngeal microbiota development will have an impact on their health in the future and in which way. 

SARS-CoV-2 RNA detection in children was associated to higher bacterial richness and increased abundance of *Fusobacterium*, *Streptococcus* and *Prevotella,* among others. To the best of our knowledge, little has been published regarding the characterization of the pediatric nasopharyngeal microbiota in the context of SARS-CoV-2 infection. Xu et al. longitudinally characterized the nasopharyngeal and the gut microbiota in nine children with SARS-CoV-2 infection [35]. Although these authors found reduced microbial richness, the predominant bacterial genera found in children were like those found in our study, except for *Pseudomonas* sp. 

Interestingly, opposite associations have been published between different *Fusobacterium* species and COVID-19 disease in adults. While *Nardelli* et al. found a reduced abundance of *Fusobacterium periodonticum* in SARS-CoV-2 infected subjects [36], Wolff et al. described cases of bacteremia caused by *Fusobacterium nucleatum* as a complication of COVID-19 disease in an elderly population [37]. 

Despite the overall low abundance of *Streptococcus* in the entire study population, a positive correlation was found between this genus and the detection of SARS-CoV-2 RNA in children. In line with our results, Aykaca et al. assessed pneumococcal carriage in children and found higher rates in those with COVID-19 than in non-infected children, yet no effects were observed in the course of COVID-19 disease [38]. 

Lastly, higher *Prevotella* abundance was also correlated with SARS-CoV-2 RNA detection in children, in agreement with previous studies which linked this bacterial genus to common viral or bacterial acute respiratory infections [10] as well as to COVID-19 [35].

Among adults, SARS-CoV-2 RNA detection itself, a measurement for viral persistence in this dataset, did not seem to deeply impact microbial richness, the overall nasopharyngeal microbiota structure or the composition of the topmost abundant bacterial genera. Subtle differences were identified instead. Although these results are in line with De Maio et al. [39], there is some controversy on whether SARS-CoV-2 infection correlates to either reduced [40,41] or not altered [39,42] nasopharyngeal microbiota diversity in adults. We found limited significant differences in taxon abundances by SARS-CoV-2 RNA detection in adults, with only the increased abundance of an unclassified member of the *Actinomycetales* order among those with persistent SARS-CoV-2 infection. However, other studies found several bacterial taxa associated to SARS-CoV-2 infection. Specifically, Mostafa et al. found lower *Corynebacterium accolens* as well as higher *Propionibacteriaceae* abundance in nasopharyngeal swabs from SARS-CoV-2-infected adults [41], but neither *Corynebacterium* or *Propionibacterium* showed differential abundance in our dataset. Engen et al. analyzed the nasopharyngeal swabs of 19 subjects in the early stage of the pandemic, which would resemble our setting, and found *Corynebacterium*, *Moraxella* and *Staphylococcus* among the six dominant taxa in both groups irrespective of SARS-CoV-2 infection [39]. While these findings agree with our results, they also reported a decreased *Corynebacterium* and *Streptococcus* abundance with COVID-19 disease, whereas these taxa were not differentially abundant in our adult dataset. Nardelli et al. reported a significantly lower abundance of *Leptotrichia*, *Fusobacterium* and *Haemophilus* among subjects with COVID-19 [36], but again, none of these were found to be altered in our adult population. It should be noted that this observed variability in COVID-19-associated changes in the respiratory microbiota in adults may be directly related to the variety in the sample type and time of sampling, lab processing procedures and quality of extracted DNA, as well as subjects’ demographic, social and clinical characteristics. It is important to bear in mind that these above-mentioned studies compared adults with current SARS-CoV-2 infection to uninfected controls, whereas in our dataset all adults reported a previous positive SARS-CoV-2-RT-PCR result, and thus our findings relate to SARS-CoV-2 RNA persistence instead of infection status.

Finally, our dataset showed an association between COVID-19 severity, represented by past COVID-19-related hospitalization, and increased *Staphylococcus* and reduced *Dolosigranulum* abundance, among others, in adults. Interestingly, previous publications described an antagonistic role between *Staphylococcus* and *Dolosigranulum* species in the respiratory tract [31]. A study from Meresntein et al. collected endotracheal aspirates from 24 COVID-19 critically-ill-intubated individuals and compared them to those of healthy controls, and found reduced diversity and an increased prevalence and abundance of *Staphylococcus*, among other common respiratory pathogens, in the former [40]. While our results also show a decreased abundance of commensals and increased pathobionts related to increased severity of COVID-19, we cannot confirm whether these changes in the nasopharynx are a cause or a consequence of COVID-19 hospitalization in our adult population. Notably, adults with past COVID-19-related hospitalization showed significantly higher antibiotic use within the three months before sample collection, and multiple previous studies have reported increased *Staphylococcus* [11] and loss of *Corynebacterium* and *Dolosigranulum* [43] associated to antibiotic use in children.

Findings reported in this study are subject to limitations. First, in our study we were very strict with the quality of participant’s samples since we used the surplus of DNA extracts previously used to detect SARS-CoV-2 RNA by RT-PCR. Consequently, many samples were excluded from the microbiota analysis either due to contamination or not enough good quality DNA for further processing. Second, we did not collect longitudinal data that could allow us to more properly assess how the nasopharyngeal microbiota evolves according to the changing policies on self-containment and social distancing measures. Third, samples from SARS-CoV-2-uninfected adults were not available for assessment, thus facing our question towards the effect of SARS-CoV-2 RNA persistence on the nasopharyngeal microbiota instead of infection itself. Moreover, when assessing the effect of COVID-19 severity on the nasopharyngeal microbiota, we could not rule out an uncontrolled effect of the antibiotic use on those subjects with past COVID-19 hospitalization. Fourth, we did not have data on cytokine profiling nor other markers of disease progression in adults, which could have provided useful information for a deeper understanding of SARS-CoV-2 pathogenesis.

## 5. Conclusions

In conclusion, COVID-19 lockdown seemed to have a remarkable impact on children’s nasopharyngeal microbiota, and future follow-up work should study the potential effect on microbiota colonization and health. Nasopharyngeal microbiota dominated by *Corynebacterium* was found both in children and adults with a low relative abundance of common pathobionts including *Streptococcus* and *Haemophilus*. In adults, persistent SARS-CoV-2 RNA detection was associated with an increased abundance of an unclassified member of the *Actinomycetales* order, whereas COVID-19 severity in adults was associated with decreased healthy commensal bacteria and increased pathobionts in their nasopharynx.

## Figures and Tables

**Table 1:** Demographics of the pediatric population by SARS-CoV-2 RNA detection by RT-PCR.

		All Infants (*n* = 470)	SARS-CoV-2 RNA Detection		*p*-Value *Pos* vs. *Neg*
			Positive (*n* = 45)	Negative (*n* = 425)	
Gender, female		226 (48.1%)	22 (48.9%)	204 (48%)	1 ^a^
Median age, years (IQR)		4.4 (2.6, 7.5)	4.7 (2.4, 8.5)	4.4 (2.7, 7.4)	0.619 ^b^
Days since adult’s infection (IQR)		52.5 (42, 61)	49 (36, 59)	53 (43, 61)	0.181 ^b^
Median body temperature, °C (IQR)		36 (35.7, 36.3)	36 (35.6, 36.2)	36 (35.7, 36.3)	0.371 ^b^
Active respiratory symptoms		21 (*n* = 468) (4.5%)	3 (*n* = 41) (6.8%)	18 (*n* = 424) (4.3%)	0.688 ^a^
Antibiotic use (last 3 months)		56 (*n* = 215) (26%)	7 (*n* = 24) (29.2%)	49 (*n* = 191) (25.7%)	0.923 ^a^
Probiotic use (last 3 months)		17 (*n* = 7.9%)	1 (*n* = 24) (4.2%)	16 (*n* = 191) (8.4%)	0.750 ^a^
Other respiratory viruses	**Overall**	115 (24.5%)	**20 (44.4%)**	**95 (22.4%)**	**0.002 ^a^**
	**Rhinovirus/Enterovirus**	89 (18.9%)	**18 (40%)**	**71 (16.7%)**	**<0.001 ^a^**
	Adenovirus	13 (2.8%)	1 (2.2%)	12 (2.8%)	1 ^a^
	Bocavirus	29 (6.2%)	6 (13.3%)	23 (5.4%)	0.076 ^a^
	Coronavirus	1 (0.21%)	0 (0%)	1 (0.24%)	1 ^c^
	Metapneumovirus	0 (0%)	0 (0%)	0 (0%)	-
	VRS (type A and B)	1 (0.21%)	0 (0%)	1 (0.24%) *	1 ^c^
	Influenza virus (A and B)	1 (0.21%)	0 (0%)	1 (0.24%) **	1 ^c^
	**Parainfluenza virus 1**	6 (1.28%)	**3 (6.7%)**	**3 (0.70%)**	**0.013 ^c^**
	Parainfluenza virus 2	4 (0.85%)	1 (2.2%)	3 (0.70%)	0.332 ^c^
	Parainfluenza virus 3	1 (0.21%)	0 (0%)	1 (0.24%)	1 ^c^
	Parainfluenza virus 4	0	0 (0%)	0 (0%) (*n* = 424)	-

^a^ Chi-square test. ^b^ Wilcoxon Rank Sum test. ^c^ Fisher exact test. Abbreviations: IQR, interquartile range. * VRS Type B, ** Influenza Virus Type A, (*n* = X) indicate the total number of subjects with available data, Values expressed as No. (%) unless otherwise stated. Continuous variables are described as median and interquartile range (IQR) values.

**Table 2:** Demographics of the adult population by SARS-CoV-2 RNA detection by RT-PCR and COVID-19 severity.

		All Adults (*n* = 173)	SARS-CoV-2 RNA Detection		*p*-Value *Pos* vs. *Neg*
			Positive (*n* = 47)	Negative (*n* = 126)	
Gender, female		63 (36.4%)	30 (63.8%)	80 (63.5%)	1 ^a^
Median age, years (IQR)		39.9 (35.9, 44.4)	40 (36.2, 45.8)	39.9 (35.9, 43.9)	0.639 ^b^
Days since first infection (IQR)		53 (44, 61)	50 (43.5, 56.5)	53.5 (46, 61)	0.150 ^b^
Median body temperature, °C (IQR)		36 (35.6, 36.2)	36.1 (35.6, 36.3)	35.9 (35.5, 36.2)	0.298 ^b^
**Active respiratory symptoms**		20 (*n* = 170) (11.8%)	**10 (*n* = 45) (22.2%)**	**10 (*n* = 125) (8%)**	**0.023 ^a^**
Antibiotic use (last 3 months)		54 (*n* = 138) (39.1%)	13 (*n* = 41) (31.7%)	41 (*n* = 97) (42.3%)	0.332 ^b^
Probiotic use (last 3 months)		15 (*n* = 138) (10.9%)	4 (*n* = 40) (10%)	11 (*n* = 98) (11.2%)	1 ^b^
Other respiratory viruses	**Overall**	10 (*n* = 172) (5.8%)	**7 (14.9%)**	**3 (*n* = 125) (2.4%)**	**0.006 ^a^**
	**Rhinovirus/Enterovirus**	9 (*n* = 172) (5.2%)	**6 (12.8%)**	**3 (*n* = 125) (2.4%)**	**0.019 ^a^**
	Adenovirus	0 (*n* = 172) (0%)	0 (0%)	0 (*n* = 125) (0%)	-
	Bocavirus	2 (*n* = 172) (1.2%)	1 (2.1%)	1 *(n* = 125) (0.8%)	0.473 ^c^
	Coronavirus	0 (*n* = 172) (0%)	0 (0%)	0 (*n* = 125) (0%)	-
	Metapneumovirus	0 (*n* = 172) (0%)	0 (0%)	0 (*n* = 125) (0%)	-
	VRS (type A and B)	0 (*n* = 172) (0%)	0 (0%)	0 (*n* = 125) (0%)	-
	Influenza virus (A and B)	1 (*n* = 172) (0.58%) *	1 (2.1%)	0 (*n* = 125) (0%)	-
	Parainfluenza virus 1	0 (*n* = 172) (0%)	0 (0%)	0 (*n* = 125) (0%)	-
	Parainfluenza virus 2	0 (*n* = 172) (0%)	0 (0%)	0 (*n* = 125) (0%)	-
	Parainfluenza virus 3	0 (*n* = 172) (0%)	0 (0%)	0 (*n* = 125) (0%)	-
	Parainfluenza virus 4	0 (*n* = 172) (0%)	0 (0%)	0 (*n* = 125) (0%)	-
	**COVID-19 inpatient history**		***p*-value** ***Yes* vs. *No***		
	**Yes (*n* = 36)**	**No (*n* = 137)**			
**Gender, female**	**11 (30.6%)**	**99 (72.3%)**	**<0.001 ^a^**		
**Median age, years (IQR)**	**44.2 (36.9, 48.4)**	**39 (35.6, 42.9)**	**<0.001 ^b^**		
Days since first infection (IQR)	51 (41.8, 56.8)	53 (46, 61)	0.255 ^b^		
Median body temperature, °C (IQR)	36 (35.6, 36.3)	36 (35.6, 36.2)	0.804 ^b^		
Active respiratory symptoms	9 (*n* = 35) (25.7%)	11 (*n* = 135) (8.1%)	0.551 ^a^		
**Antibiotic use (last 3 months)**	**29 (*n* = 34) (85.3%)**	**25 (*n* = 104) (24%)**	**<0.001 ^a^**		
Probiotic use (last 3 months)	6 (*n* = 33) (18.2%)	9 (*n* = 105) (8.6%)	0.22 ^a^		
Actual SARS-CoV-2 PCR (positive)	8 (22.2%)	39 (28.5%)	0.589 ^a^		
Other respiratory viruses	Overall	1 (*n* = 35) (2.9%)	9 (6.60%)	0.665 ^a^	
	Rhinovirus/Enterovirus	1 (*n* = 35) (2.9%)	8 (5.84%)	0.778 ^a^	
	Adenovirus	0 (*n* = 35) (0%)	0 (0%)	-	
	Bocavirus	0 (*n* = 35) (0%)	2 (1.46%)	1 ^c^	
	Coronavirus	0 (*n* = 35) (0%)	0 (0%)	-	
	Metapneumovirus	0 (*n* = 35) (0%)	0 (0%)	-	
	VRS (type A and B)	0 (*n* = 35) (0%)	0 (0%)	-	
	Influenza virus (A and B)	0 (*n* = 35) (0%)	1 (0.73%) *	1 ^c^	
	Parainfluenza virus 1	0 (*n* = 35) (0%)	0 (0%)	-	
	Parainfluenza virus 2	0 (*n* = 35) (0%)	0 (0%)	-	
	Parainfluenza virus 3	0 (*n* = 35) (0%)	0 (0%)	-	
	Parainfluenza virus 4	0 (*n* = 35) (0%)	0 (0%)	-	

^a^ Chi-square test. ^b^ Wilcoxon Rank Sum test. ^c^ Fisher exact test. Abbreviations: IQR, interquartile range, * Influenza Virus Type B, (n = X) indicate the total number of subjects with available data, values expressed as No. (%) unless otherwise stated. Continuous variables are described as median and interquartile range (IQR) values.

## Data Availability

The datasets generated during and/or analyzed during the current study are available in the European Nucleotide Archive (ENA) repository (https://www.ebi.ac.uk/ena/browser/home accessed on 4 March 2022) under Bioproject PRJEB51337. Code used for the bioinformatic and statistical analysis and plotting the data (https://figshare.com/s/69d94ea739860f3857a9 accessed on 1 March 2022) as well as the input data file (https://figshare.com/s/22e2ce6a1d980c566a3a accessed on 20 January 2022) and color dictionary (https://figshare.com/s/3fd77774906307c34e6c accessed on 1 March 2022) have been uploaded to FigShare.

## Supplementary Materials

The following supporting information can be downloaded at: https://www.mdpi.com/article/10.3390/v14071521/s1, Table S1: Epidemiological, clinical, and microbiological characteristics of participants younger than 5 split by SARS-CoV-2 RNA detection by RT-PCR; Table S2: Epidemiological, clinical, and microbiological characteristics of participants older than 5 split by SARS-CoV-2 RNA detection by RT-PCR; Figure S1: Bacterial genera composition in the nasopharynx of children split by age range; Figure S2: Bacterial nasopharyngeal microbiota composition in adults split by SARS-CoV-2 persistent infection and COVID-19 severity.

## Other Kids Corona Study Group Members

Cristina Adroher, Laia Alsina, Sara Ajanovic, Ainhoa Andueza, Barbara Baro, Elisenda Bonet-Carne, Elena Buetas, Daniel Cuadras, Marta Cubells, Claudia Fortuny, Aleix Garcia-Miguel, Monica Girona, Maria Hernandez, Monica Morales, Felipe Pérez-Soler, María Ríos, Gemma Pons, Clara Prats, Francesc Torrents, Iris Uribesalgo, Anna Valls and Rosauro Varo.

## References

[1] Zhu N., Zhang D., Wang W., Li X., Yang B., Song J., Zhao X., Huang B., Shi W., Lu R. (2020). A Novel Coronavirus from Patients with Pneumonia in China, 2019. N. Engl. J. Med..

[2] Rosenwald M.S. History’s Deadliest Pandemics: Plague, Smallpox, Flu, Covid-19. https://www.washingtonpost.com/graphics/2020/local/retropolis/coronavirus-deadliest-pandemics/.

[3] Government Decrees State of Emergency to Stop Spread of Coronavirus COVID-19 [Government/Council of Ministers]. https://www.lamoncloa.gob.es/lang/en/gobierno/councilministers/Paginas/2020/20200314council-extr.aspx.

[4] Ahmed F., Zviedrite N., Uzicanin A. (2018). Effectiveness of workplace social distancing measures in reducing influenza transmission: A systematic review. BMC Public Health.

[5] MacIntyre R.C., Chughta A.A. (2015). Facemasks for the prevention of infection in healthcare and community settings. BMJ.

[6] Brueggemann A.B., Jansen van Rensburg M.J., Shaw D., McCarthy N.D., Jolley K.A., Maiden M.C., van der Linden M.P., Amin-Chowdhury Z., Bennett D.E., Borrow R. (2021). Changes in the incidence of invasive disease due to Streptococcus pneumoniae, Haemophilus influenzae, and Neisseria meningitidis during the COVID-19 pandemic in 26 countries and territories in the Invasive Respiratory Infection Surveillance Initiative: A prospective analysis of surveillance data. Lancet. Digit. Health.

[7] Haak B.W., Brands X., Davids M., Peters-Sengers H., Kullberg R.F.J., van Houdt R., Hugenholtz F., Faber D.R., Zaaijer H.L., Scicluna B.P. (2021). Bacterial and viral respiratory tract microbiota and host characteristics in adults with lower respiratory tract infections: A case-control study. Clin. Infect. Dis..

[8] Lanaspa M., Bassat Q., Medeiros M.M., Muñoz-Almagro C. (2017). Respiratory microbiota and lower respiratory tract disease. Expert Rev. Anti. Infect. Ther..

[9] de Steenhuijsen Piters W.A.A., Sanders E.A.M., Bogaert D. (2015). The role of the local microbial ecosystem in respiratory health and disease. Philos. Trans. R. Soc. B Biol. Sci..

[10] Henares D., Brotons P., de Sevilla M.F., Fernandez-Lopez A., Hernandez-Bou S., Perez-Argüello A., Mira A., Muñoz-Almagro C., Cabrera-Rubio R. (2021). Differential nasopharyngeal microbiota composition in children according to respiratory health status. Microb. Genom..

[11] Henares D., Rocafort M., Brotons P., de Sevilla M.F., Mira A., Launes C., Cabrera-Rubio R., Muñoz-Almagro C. (2021). Rapid Increase of Oral Bacteria in Nasopharyngeal Microbiota After Antibiotic Treatment in Children With Invasive Pneumococcal Disease. Front. Cell. Infect. Microbiol..

[12] Man W.H., De Steenhuijsen Piters W.A.A., Bogaert D. (2017). The microbiota of the respiratory tract: Gatekeeper to respiratory health. Nat. Rev. Microbiol..

[13] Brotons P., Launes C., Buetas E., Fumado V., Henares D., de Sevilla M.F., Redin A., Fuente-Soro L., Cuadras D., Mele M. (2021). Susceptibility to Severe Acute Respiratory Syndrome Coronavirus 2 Infection Among Children and Adults: A Seroprevalence Study of Family Households in the Barcelona Metropolitan Region, Spain. Clin. Infect. Dis..

[14] Callahan B.J., McMurdie P.J., Rosen M.J., Han A.W., Johnson A.J.A., Holmes S.P. (2016). DADA2: High-resolution sample inference from Illumina amplicon data. Nat. Methods.

[15] Cole J.R., Wang Q., Fish J.A., Chai B., McGarrell D.M., Sun Y., Brown C.T., Porras-Alfaro A., Kuske C.R., Tiedje J.M. (2014). Ribosomal Database Project: Data and tools for high throughput rRNA analysis. Nucleic Acids Res..

[16] McMurdie P.J., Holmes S. (2013). phyloseq: An R package for reproducible interactive analysis and graphics of microbiome census data. PLoS ONE.

[17] Oksanen J., Blanchet F.G., Kindt R., Legendre P., Minchin P.R., O’Hara R.B., Simpson G.L., Solymos P., Stevens M.H.H., Wagner H. (2013). Package ‘vegan’. R Package Version 2.0–8.

[18] Lin H., Das Peddada S. (2020). Analysis of compositions of microbiomes with bias correction. Nat. Commun..

[19] Davis N.M., Proctor D.M., Holmes S.P., Relman D.A., Callahan B.J. (2018). Simple statistical identification and removal of contaminant sequences in marker-gene and metagenomics data. Microbiome.

[20] Camelo-Castillo A., Henares D., Brotons P., Galiana A., Rodríguez J.C., Mira A., Muñoz-Almagro C. (2019). Nasopharyngeal Microbiota in Children With Invasive Pneumococcal Disease: Identification of Bacteria With Potential Disease-Promoting and Protective Effects. Front. Microbiol..

[21] Bosch A.A.T.M., de Steenhuijsen Piters W.A.A., van Houten M.A., Chu M.L.J.N., Biesbroek G., Kool J., Pernet P., de Groot P.K.C.M., Eijkemans M.J.C., Keijser B.J.F. (2017). Maturation of the Infant Respiratory Microbiota, Environmental Drivers, and Health Consequences. A Prospective Cohort Study. Am. J. Respir. Crit. Care Med..

[22] Bogaert D., Keijser B., Huse S., Rossen J., Veenhoven R., van Gils E., Bruin J., Montijn R., Bonten M., Sanders E. (2011). Variability and Diversity of Nasopharyngeal Microbiota in Children: A Metagenomic Analysis. PLoS ONE.

[23] Biesbroek G., Bosch A.A.T.M., Wang X., Keijser B.J.F., Veenhoven R.H., Sanders E.A.M., Bogaert D. (2014). The impact of breastfeeding on nasopharyngeal microbial communities in infants. Am. J. Respir. Crit. Care Med..

[24] Bosch A.A.T.M., Levin E., van Houten M.A., Hasrat R., Kalkman G., Biesbroek G., de Steenhuijsen Piters W.A.A., de Groot P.K.C.M., Pernet P., Keijser B.J.F. (2016). Development of Upper Respiratory Tract Microbiota in Infancy is Affected by Mode of Delivery. EBioMedicine.

[25] Kaul D., Rathnasinghe R., Ferres M., Tan G.S., Barrera A., Pickett B.E., Methe B.A., Das S.R., Budnik I., Halpin R.A. (2020). Microbiome disturbance and resilience dynamics of the upper respiratory tract during influenza A virus infection. Nat. Commun..

[26] Dubourg G., Edouard S., Raoult D. (2019). Relationship between nasopharyngeal microbiota and patient’s susceptibility to viral infection. Expert Rev. Anti. Infect. Ther..

[27] Teo S.M., Mok D., Pham K., Kusel M., Serralha M., Troy N., Holt B.J., Hales B.J., Walker M.L., Hollams E. (2015). The infant nasopharyngeal microbiome impacts severity of lower respiratory infection and risk of asthma development. Cell Host Microbe.

[28] Buehrle D.J., Wagener M.M., Nguyen M.H., Clancy C.J. (2021). Trends in Outpatient Antibiotic Prescriptions in the United States During the COVID-19 Pandemic in 2020. JAMA Netw. Open.

[29] Peñalva G., Benavente R.S., Pérez-Moreno M.A., Pérez-Pacheco M.D., Pérez-Milena A., Murcia J., Cisneros J.M. (2021). Effect of the coronavirus disease 2019 pandemic on antibiotic use in primary care. Clin. Microbiol. Infect..

[30] Bomar L., Brugger S.D., Yost B.H., Davies S.S., Lemon K.P. (2016). Corynebacterium accolens Releases Antipneumococcal Free Fatty Acids from Human Nostril and Skin Surface Triacylglycerols. MBio.

[31] Brugger S.D., Eslami S.M., Pettigrew M.M., Escapa I.F., Henke M.T., Kong Y., Lemon K.P., D’orazio S.E.F. (2020). Dolosigranulum pigrum Cooperation and Competition in Human Nasal Microbiota. mSphere.

[32] Cremers A.J., Zomer A.L., Gritzfeld J.F., Ferwerda G., van Hijum S.A., Ferreira D.M., Shak J.R., Klugman K.P., Boekhorst J., Timmerman H.M. (2014). The adult nasopharyngeal microbiome as a determinant of pneumococcal acquisition. Microbiome.

[33] De Boeck I., Wittouck S., Wuyts S., Oerlemans E.F.M., van den Broek M.F.L., Vandenheuvel D., Vanderveken O., Lebeer S. (2017). Comparing the healthy nose and nasopharynx microbiota reveals continuity as well as niche-specificity. Front. Microbiol..

[34] Burchill E., Lymberopoulos E., Menozzi E., Budhdeo S., McIlroy J.R., Macnaughtan J., Sharma N. (2021). The Unique Impact of COVID-19 on Human Gut Microbiome Research. Front. Med..

[35] Xu R., Liu P., Zhang T., Wu Q., Zeng M., Ma Y., Jin X., Xu J., Zhang Z., Zhang C. (2021). Progressive deterioration of the upper respiratory tract and the gut microbiomes in children during the early infection stages of COVID-19. J. Genet. Genom..

[36] Nardelli C., Gentile I., Setaro M., Di Domenico C., Pinchera B., Buonomo A.R., Zappulo E., Scotto R., Scaglione G.L., Castaldo G. (2021). Nasopharyngeal Microbiome Signature in COVID-19 Positive Patients: Can We Definitively Get a Role to Fusobacterium periodonticum?. Front. Cell. Infect. Microbiol..

[37] Wolff L., Martiny D., Deyi V.Y.M., Maillart E., Clevenbergh P., Dauby N. (2021). COVID-19–Associated Fusobacterium nucleatum Bacteremia, Belgium. Emerg. Infect. Dis..

[38] Aykac K., Ozsurekci Y., Cura Yayla B.C., Evren K., Lacinel Gurlevik S., Oygar P.D., Yucel M., Karakoc A.E., Alp A., Cengiz A.B. (2021). Pneumococcal carriage in children with COVID-19. Hum. Vaccines Immunother..

[39] Engen P.A., Naqib A., Jennings C., Green S.J., Landay A., Keshavarzian A., Voigt R.M. (2021). Nasopharyngeal Microbiota in SARS-CoV-2 Positive and Negative Patients. Biol. Proced. Online.

[40] Merenstein C., Liang G., Whiteside S.A., Cobián-Güemes A.G., Merlino M.S., Taylor L.J., Glascock A., Bittinger K., Tanes C., Graham-Wooten J. (2021). Signatures of COVID-19 severity and immune response in the respiratory tract microbiome. medRxiv.

[41] Mostafa H.H., Fissel J.A., Fanelli B., Bergman Y., Gniazdowski V., Dadlani M., Carroll K.C., Colwell R.R., Simner P.J. (2020). Metagenomic Next-Generation Sequencing of Nasopharyngeal Specimens Collected from Confirmed and Suspect COVID-19 Patients. MBio.

[42] De Maio F., Posteraro B., Ponziani F.R., Cattani P., Gasbarrini A., Sanguinetti M. (2020). Nasopharyngeal Microbiota Profiling of SARS-CoV-2 Infected Patients. Biol. Proced. Online.

[43] Pettigrew M.M., Laufer A.S., Gent J.F., Kong Y., Fennie K.P., Metlay J.P. (2012). Upper Respiratory Tract Microbial Communities, Acute Otitis Media Pathogens, and Antibiotic Use in Healthy and Sick Children. Appl. Environ. Microbiol..

