# Supplementary material for: Impact of COVID-19 Lockdown on the Nasopharyngeal Microbiota of Children and Adults Self-Confined at Home
Source: Viruses. 2022 Jul 12;14(7):1521. doi: 10.3390/v14071521 (PMC9315980; doi:10.3390/v14071521)

Supplementary Table S 1. Epidemiological, clinical, and microbiological characteristics of participants younger than 5 split by SARS-CoV-2 RNA detection by RT-PCR. Values expressed as No. (%), unless otherwise stated. Continuous variables were described as median and interquartile range (IQR) values.

		**All infants ≤ 5 years (*n*=265)**	**SARS-CoV-2 RNA detection**		**P value**
			**Positive (*n*=24)**	**Negative (*n*=241)**	*Pos vs. Neg*
Gender, female		115 (43.4%)	8 (33.3%)	107 (44.4%)	0.41[a]
Median age, years (IQR)		2.9 (1.8, 3.8)	2.5 (1.7, 3.8)	2.9 (1.8, 3.8)	0.728[b]
Days since adult's infection (IQR)		53 (43, 62)	47 (39, 59.5)	53 (44, 62)	0.057[b]
Median body temperature, ºC (IQR)		36.1 (35.8, 36.4)	36 (25.8, 36.2)	36.1 (35.8, 36.4)	0.622[b]
Active respiratory symptoms		9 (3.4%)	1 (4.2%)	8 (3.3%)	1[a]
Antibiotic use (last 3 months)		37 (*n*=138) (26.8%)	5 (*n*=15) (33.3%)	32 (*n*=123) (26%)	0.768[a]
Probiotic use (last 3 months)		10 (*n*=137) (7.3%)	1 (*n*=15) (6.7%)	9 (*n*=122) (7.4%)	1[a]
Other respiratory viruses	**Overall**	**83 (31.3%)**	**14 (58.3%)**	**69 (28.6%)**	**0.006[a]**
	**Rhinovirus/Enterovirus**	**60 (22.6%)**	**12 (50%)**	**48 (19.9%)**	**0.002[a]**
	Adenovirus	12 (4.5%)	1 (4.2%)	11 (4.6%)	1[a]
	**Bocavirus**	**28 (10.6%)**	**6 (25%)**	**22 (9.1%)**	**0.039[a]**
	Coronavirus	1 (0.38%)	0 (0%)	1 (0.42%)	1[a]
	Metapneumovirus	0 (0%)	0 (0%)	0 (0%)	-
	VRS (type A and B)	1 (0.38%)	0 (0%)	1 (0.42%)*	1[a]
	Influenza virus (A and B)	0 (0%)	0 (0%)	0 (0%)	-
	**Parainfluenza virus 1**	**5 (1.9%)**	**3 (12.5%)**	**2 (0.83%)**	**0.006[c]**
	Parainfluenza virus 2	3 (1.1%)	1 (4.2%)	2 (0.83%)	0.248[c]
	Parainfluenza virus 3	1 (0.38%)	0 (0%)	1 (0.42%)	1[a]
	Parainfluenza virus 4	0 (0%)	0 (0%)	0 (0%)	-

[a]Chi-square test.
[b]Wilcoxon Rank Sum test.
[c]Fisher exact test.
Abbreviations: IQR, Interquartile Range
*VRS Type B
(*n*=X) indicate the total number of subjects with available data

Supplementary Table S 2. Epidemiological, clinical, and microbiological characteristics of participants older than 5 split by SARS-CoV-2 RNA detection by RT-PCR. Values expressed as No. (%), unless otherwise stated. Continuous variables were described as median and interquartile range (IQR) values.

		All infants > 5 years (*n*=205)	SARS-CoV-2 RNA detection		P value
			Positive (*n*=21)	Negative (*n*=184)	*Pos vs. Neg*
Gender, female		94 (45.9%)	14 (66.7%)	97 (52.7%)	0.325[a]
Median age, years (IQR)		8.2 (6.4, 10.7)	9.4 (6.9, 12.3)	8.1 (6.4, 10.4)	0.365[b]
Days since adult's infection (IQR)		52 (41, 59)	54 (34, 59)	51 (42, 59)	0.956[b]
Median body temperature, ºC (IQR)		36 (35.6, 36.2)	35.8 (35.5, 36.2)	36 (35.7, 36.2)	0.373[b]
Active respiratory symptoms		12 (*n*=203) (5.9%)	2 (*n*=20) (10%)	10 (*n*=183) (5.5%)	0.751[a]
Antibiotic use (last 3 months)		19 (*n*=77) (24.7%)	2 (*n*=9) (22.2%)	17 (*n*=68) (25%)	1[a]
Probiotic use (last 3 months)		7 (*n*=77) (9.1%)	0 (*n*=9) (0%)	7 (*n*=69) (10.1%)	0.703[a]
Other respiratory viruses	Overall	32 (15.6%)	6 (28.6%)	26 (14.1%)	0.159[a]
	Rhinovirus/Enterovirus	29 (14.1%)	6 (28.6%)	23 (12.5%)	0.094[a]
	Adenovirus	1 (0.49%)	0 (0%)	1 (0.54%)	1[c]
	Bocavirus	1 (0.49%)	0 (0%)	1 (0.54%)	1[c]
	Coronavirus	0 (0%)	0 (0%)	0 (0%)	-
	Metapneumovirus	0 (0%)	0 (0%)	0 (0%)	-
	VRS (type A and B)	0 (0%)	0 (0%)	0 (0%)	-
	Influenza virus (A and B)	1 (0.49%)	0 (0%)	1 (0.54%)*	1[c]
	Parainfluenza virus 1	1 (0.49%)	0 (0%)	1 (0.54%)	1[c]
	Parainfluenza virus 2	1 (0.49%)	0 (0%)	1 (0.54%)	1[c]
	Parainfluenza virus 3	0 (0%)	0 (0%)	0 (0%)	-
	Parainfluenza virus 4	0 (*n*=204) (0%)	0 (0%)	0 (*n*=183) (0%)	-

[a]Chi-square test.
[b]Wilcoxon Rank Sum test.
[c]Fisher exact test.
Abbreviations: IQR, Interquartile Range
*Influenza Virus Type A
(*n*=X) indicate the total number of subjects with available data

Supplementary Figure S1. Bacterial genera composition in the nasopharynx of children split by age range. Bacterial genera were filtered by a minimum of 0.01% relative abundance in at least 10% of samples within each study group (children <=5 years in A and children > 5 years in B). Only bacterial genera with a mean abundance >1% are shown in the table ranked from most to least abundant in each group. Same genera are properly identified by color coding as shown in the legend and kept consistent in the two groups. Bacterial genera whose mean relative abundance was <1% are grouped into “others”.

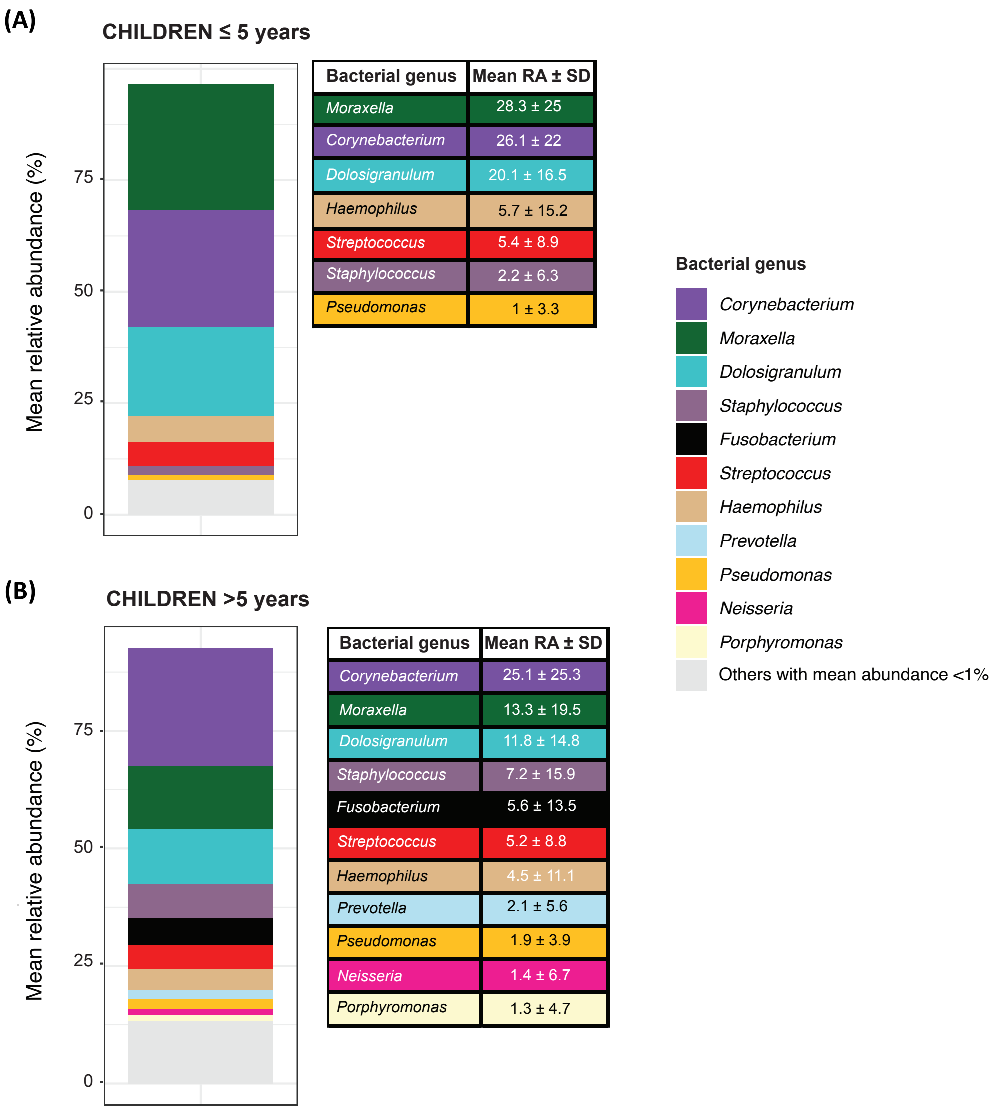

(A) CHILDREN ≤ 5 years

Bacterial genus	Mean RA ± SD
Moraxella	28.3 ± 25
Corynebacterium	26.1 ± 22
Dolosigranulum	20.1 ± 16.5
Haemophilus	5.7 ± 15.2
Streptococcus	5.4 ± 8.9
Staphylococcus	2.2 ± 6.3
Pseudomonas	1 ± 3.3

(B) CHILDREN >5 years

Bacterial genus	Mean RA ± SD
Corynebacterium	25.1 ± 25.3
Moraxella	13.3 ± 19.5
Dolosigranulum	11.8 ± 14.8
Staphylococcus	7.2 ± 15.9
Fusobacterium	5.6 ± 13.5
Streptococcus	5.2 ± 8.8
Haemophilus	4.5 ± 11.1
Prevotella	2.1 ± 5.6
Pseudomonas	1.9 ± 3.9
Neisseria	1.4 ± 6.7
Porphyromonas	1.3 ± 4.7

Supplementary Figure S2. Bacterial nasopharyngeal microbiota composition in adults split by SARS-CoV-2 persistent infection and COVID-19 severity. Bacterial genera were filtered by a minimum of 0.01% relative abundance in at least 10% of samples within each study group (split by SARS-CoV-2 persistent RNA detection in A) and by history of COVID-19 related hospitalization in B). Only bacterial genera with a mean abundance >1% are shown in the table ranked from most to least abundant in each group. Same genera are properly identified by color coding as shown in the legend and kept consistent in the two groups. Bacterial genera whose mean relative abundance was <1% are grouped into “others”.

(A)

SARS-CoV-2-RT-PCR +

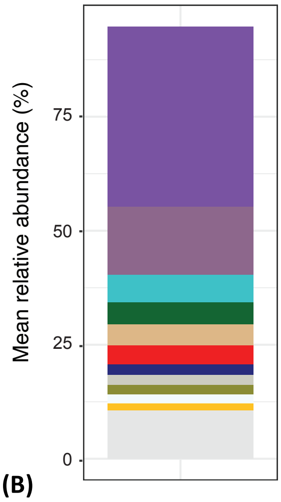

Bacterial genus	Mean RA ± SD
Corynebacterium	39.5 ± 29.5
Staphylococcus	15 ± 19.1
Dolosigranulum	6.0 ± 8.7
Moraxella	4.9 ± 13
Haemophilus	4.6 ± 13.4
Streptococcus	4.1 ± 9.1
Anaerococcus	2.4 ± 3.4
Actinomycetales	2.2 ± 3.5
Propionibacterium	2.0 ± 2.5
Peptoniphilus	2.0 ± 4.8
Pseudomonas	1.5 ± 3.6

SARS-CoV-2-RT-PCR -

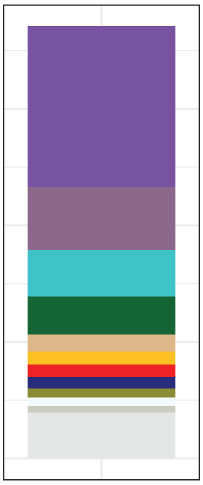

Bacterial genus	Mean RA ± SD
Corynebacterium	34.5 ± 9.1
Staphylococcus	13.6 ± 18.2
Dolosigranulum	9.9 ± 14.2
Moraxella	8.2 ± 13.8
Haemophilus	3.6 ± 12.2
Pseudomonas	2.8 ± 9.1
Streptococcus	2.7 ± 5.9
Anaerococcus	2.4 ± 4.0
Propionibacterium	2.0 ± 2.7
Peptoniphilus	1.8 ± 2.6
Actinomycetales	1.4 ± 3.4

(B)

Past COVID-19 hospitalization

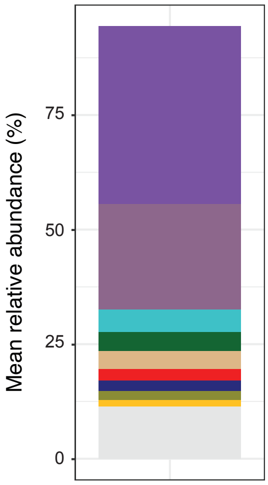

Bacterial genus	Mean RA ± SD
Corynebacterium	38.8 ± 27.7
Staphylococcus	23.1 ± 23.5
Dolosigranulum	4.9 ± 9.6
Moraxella	4.2 ± 9.2
Haemophilus	3.9 ± 13.3
Streptococcus	2.5 ± 3.9
Anaerococcus	2.3 ± 3.7
Propionibacterium	2.0 ± 2.5
Pseudomonas	1.4 ± 2.2

No hospitalization

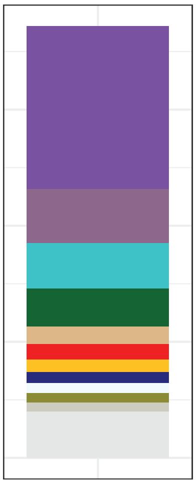

Bacterial genus	Mean RA ± SD
Corynebacterium	35.1± 25.6
Staphylococcus	11.6 ± 16.1
Dolosigranulum	9.9 ± 13.6
Moraxella	8.1 ± 14.5
Haemophilus	3.9 ± 12.3
Streptococcus	3.3 ± 7.5
Pseudomonas	2.7± 8.9
Anaerococcus	2.4 ± 3.9
Peptoniphilus	2.1 ± 4.3
Propionibacterium	2.0 ± 2.6
Actinomycetales	1.9 ± 3.7

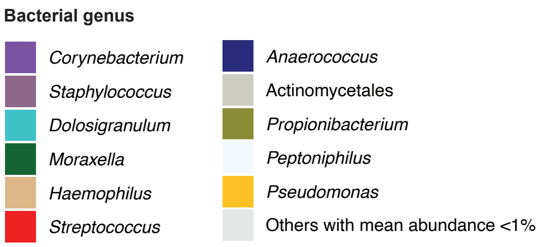

Supplement: Supplementary file 1 [file viruses-14-01521-s001.zip › viruses-1796220-supplementary.pdf]
